# Retinoid X receptor γ interacts with peroxisome proliferator-activated receptor-γ to promote browning during adipose tissue differentiation

**DOI:** 10.1080/21623945.2025.2548780

**Published:** 2025-08-25

**Authors:** Defei Chen, Saed Woraikat, Xiong Guo, Fuyu Yang, Chenglin Tang, Fan He, Kun Qian

**Affiliations:** a Department of Gastrointestinal Surgery, The First Affiliated Hospital of Chongqing Medical University, Chongqing, China; b Department of Hepatobiliary Surgery, The Second Affiliated Hospital of Chongqing Medical University, Chongqing, China

**Keywords:** RXRγ, PPARγ, adipogenesis, cofactor, brown adipocyte

## Abstract

Obesity and type 2 diabetes mellitus are global public health challenges. Activating thermogenic adipose tissues, such as brown adipose tissue and beige adipose tissue, could be a promising strategy to combat obesity and consequently obesity-related diabetes. Both peroxisome proliferator-activated receptor-γ (PPARγ) and retinoid X receptor γ (RXRγ) play significant roles in the regulation of adipogenic differentiation. However, the underlying mechanisms and interactions between these receptors during adipogenic differentiation remain unclear. In this study, we conducted a comprehensive analysis of a transcriptome sequencing dataset sourced from the GEO database, encompassing samples of white and brown adipose tissues from 15 healthy individuals. Our findings reveal that RXRγ expression is significantly elevated in brown adipose tissue relative to white adipose tissue (*p* = 0.041). Furthermore, co-immunoprecipitation assays validated that RXRγ can be co-precipitated with PPARγ. Subsequent luciferase assays demonstrated that the interaction between RXRγ and PPARγ significantly enhances the transcriptional activity of uncoupling protein 1 (UCP1) compared to the overexpression of PPARγ alone (3.4-fold vs. 1.5-fold, *p* < 0.001). Notably, in human preadipocytes, the co-overexpression of RXRγ with PPARγ resulted in a significant increase in UCP1 transcriptional activity compared to the overexpression of PPARγ alone (3.4-fold vs. 2.0-fold, *p* < 0.05). In summary, our findings suggest that RXRγ serves as a novel cofactor for PPARγ, promoting the browning of adipose tissue through the upregulation of UCP1 transcription.

## Introduction

Obesity and type 2 diabetes mellitus (T2DM) are global public health challenges [1,2]. Controlling obesity is crucial for the prevention and treatment of T2DM [3,4]. Obesity develops when energy intake exceeds expenditure. In mammals, two major types of adipose tissue exist: white adipose tissue (WAT) and brown adipose tissue (BAT). WAT stores energy in the form of triglycerides, while BAT converts energy into heat through non-shivering thermogenesis and contributes to metabolic homoeostasis. Recent studies have discovered that activating thermogenic adipose tissues, such as BAT and beige adipose tissues, could be a promising strategy to combat obesity and obesity-related diabetes [5,6]. Therefore, exploring the underlying molecular mechanisms of adipose tissue is of great significance for disease treatment.

Adipose tissue is essential for maintaining energy and metabolic homoeostasis in the body. It is not only a major organ for maintaining energy balance but also an active endocrine system, secreting various cytokines and hormones that affect cell and tissue function. The normal development of adipose tissue is key to physical health [7,8]. Brown adipocytes, the functional component of BAT, are characterized by the presence of multilocular lipid droplets, an abundance of mitochondria, and the expression of the cell type-specific uncoupling protein 1 (UCP1). Both an increase in the number of brown adipocytes and the induction of UCP1 expression are characteristics of adipocyte ‘browning’ [9].

The pathophysiology of obesity and T2DM is associated with abnormalities in endocrine signalling within adipose tissue. One of the most important signalling factors in these disorders is the nuclear hormone transcription factor peroxisome proliferator-activated receptor-γ (PPARγ) [10,11]. PPARγ is a major transcription factor in adipocyte differentiation, regulating the expression of genes related to lipid synthesis and metabolism, and playing a significant role in the production of brown fat [12]. Retinoid X receptor γ (RXRγ), a member of the nuclear receptor superfamily, is expressed in brown adipocytes, with increased gene expression observed during brown adipogenesis in stem cells [13]. Both PPARγ and RXRγ play crucial roles in the control of adipogenic differentiation. However, the underlying mechanisms and interactions between them during adipogenic differentiation remain unclear.

In this study, we found that RXRγ is highly expressed in brown adipose tissue compared to white adipose tissue. Additionally, through co-immunoprecipitation analysis, we discovered that RXRγ can be precipitated by PPARγ. Furthermore, we found that the interaction between RXRγ and PPARγ enhances the transcriptional activity of uncoupling protein 1 (UCP1), as demonstrated by a luciferase assay. In summary, our findings suggest that RXRγ is a novel cofactor for PPARγ, promoting the browning of adipose tissue by enhancing UCP1 transcription.

## Results

### RXRγ was high expression in human brown adipose tissue

**Figure 1.**
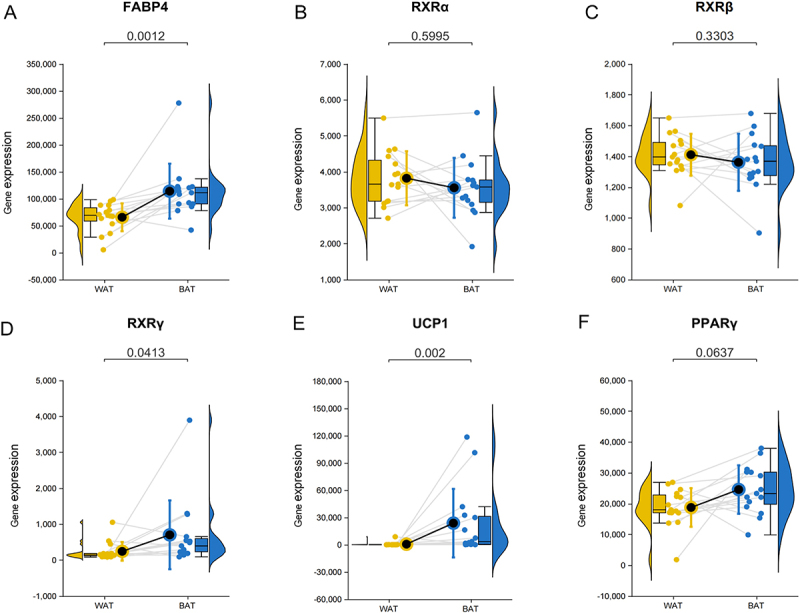
RXRγ highly expressed in browning adipose tissue. Gene expression analysis of adipose tissue-related genes in white adipose tissue (WAT) and brown adipose tissue (BAT) from healthy subjects (n = 15). (A-G) the gene expression levels of FABP4, RXRα, RXRβ, RXRγ, UCP1 and PPARγ. Data are shown as mean ± SEM and *p* value.

To identify specific factors that regulate adipose tissue browning, a transcriptome sequencing (RNA-seq) dataset (GSE113764) from the Gene Expression Omnibus (GEO) database was utilized and the coding transcript abundance of interested gene in paired biopsies of WAT and BAT obtained from the supraclavicular region of 15 healthy subjects was analysed. As shown in Figure 1. Our results indicated that the expression levels of RXRα, RXRβ, and PPARγ did not significantly differ between WAT and BAT. However, we detected tissue-specific differences in the expression levels of fatty acid-binding protein 4 (FABP4) (*p* = 0.0012), RXRγ (*p* = 0.0413) and UCP1 (*p* = 0.002), with both being significantly higher in BAT. This finding suggests that RXRγ and UCP1 may play distinct roles in the metabolic functions of these adipose tissues, contributing to the enhanced metabolic activity and thermogenesis characteristic of BAT.

### RXRγ is a PPARγ-interacting protein

To investigate the interaction between PPARγ and RXRγ, proteomics analyses were conducted first. Human preadipocytes were infected with lentiviruses expressing Flag-PPARγ or Flag-EGFP and treated the cells with rosiglitazone (Rosi) and insulin. Twenty-four hours after treatment, PPARγ-associated proteins were isolated using anti-Flag affinity beads and proteomics analyses were performed using the Flag-bound proteins. As shown in Table 1, several proteins were identified to bind to Flag-PPARγ but not to Flag-EGFP. Followed chromatography was performed in independent experiments. As expected, the most abundant protein isolated from the anti-Flag magnetic beads is PPARγ, given it is Flag-tagged and overexpressed as a bait.

**Table 1.** List of associated proteins.

Proteins (bound)	Flag-PPARγ (#uni. peps)	Flag-EGFP (#uni. peps)
PPARγ	61	0
STK38	25	0
RXRγ	17	0
STK38L	13	0
PRMT5	7	0
TCP1	4	0
MEP50	3	0

The number (#: the mean of three independent experiments) of unique peptides (uni. peps) eluted from Flag-PPARγ or Flag-EGFP affinity beads. PPARγ, peroxisome proliferator-activated receptor-γ; STK38, serine/threonine kinase 38; RXRγ, retinoid X receptor γ; STK38L, STK38-Like; PRMT5, protein arginine methyltransferase 5; TCP1, t-complex 1; MEP50, methylosome protein 50.

**Figure 2.**
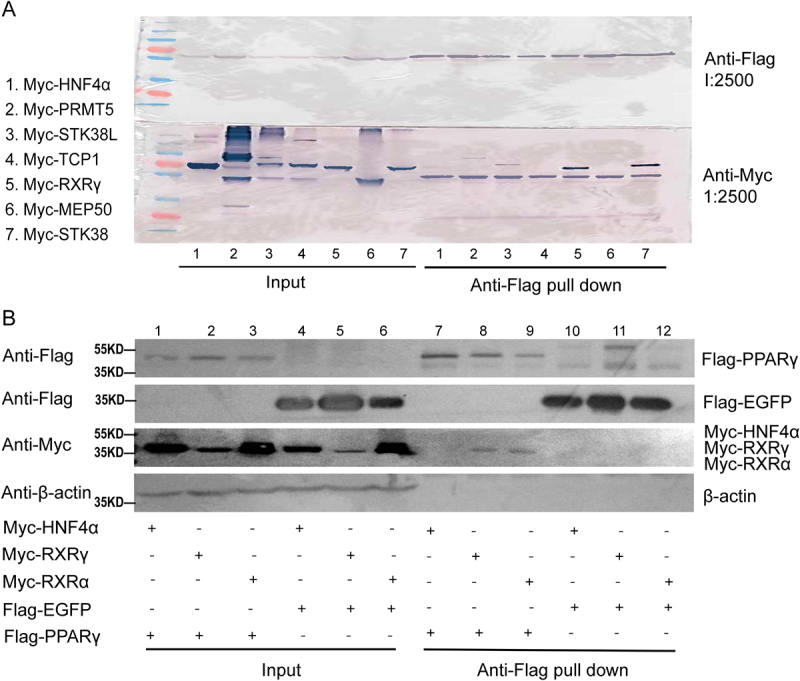
RXRγ is a PPARγ-interacting protein (A) immunoprecipitation and western blot analysis of Flag-tagged PPARγ and Myc-tagged proteins. HEK293 cells were cotransfected with Flag-PPARγ and one of the PPARγ-associated proteins. The blots were probed with anti-Flag (to detect flag-tagged PPARγ) and anti-Myc (to detect the Myc-tagged interaction partners), each at a dilution of 1:2500, the top panel shows the anti-Flag and the bottom panel shows the anti-Myc western blot. (B) immunoprecipitation and western blot analysis to detect the interactions between Flag-PPARγ or Flag-EGFP and various Myc-tagged proteins. HEK293 cells were transfected with tagged PPARγ, HNF4α (negative control), RXRγ, EGFP (negative control), or RXRα (positive control). The left side of the panel shows input levels of proteins in cell lysates before pull-down (lanes 1–6), and the right side shows proteins after anti-Flag pull-down (lanes 7–12), with β-actin serves as a loading control. The resin and immunoblotted antibodies are labelled on the left of the blot and the detected proteins are labelled on the right of the blot. The presence or absence of each protein in the assays is indicated at the bottom of the panel (+ for presence, - for absence).

To further verify the interaction between PPARγ and RXRγ, immunoprecipitation experiments were conducted (Figure 2). In the first experiment, HEK293 cells were transfected with labelled Flag-PPARγ, along with one of the PPARγ-associated proteins identified in the proteomics analyses or the control protein Myc-HNF4α. As shown in Figure 2(A), Flag-PPARγ did not pull down Myc-HNF4α, Myc-PRMT5, Myc-TCP1, or Myc-MEP50 (lane 1, 2, 4, 6). However, Myc-RXRγ (lane 6), as well as Myc-STK38 (lane 7) and Myc-STK38L (lane 3), which were proved as the cofactor of PPARγ [14], were significantly pulled down by Flag-PPARγ. In the second experiment, Anti-Flag pull-down experiments were performed in HEK293 cells by co-transfecting labelled Myc-RXRγ and Flag-PPARγ, along with control proteins Myc-RXRα, Flag-EGFP and Myc-HNF4α. Flag-EGFP and Myc-HNF4α are nuclear transcription factors previously shown not to bind PPARγ and Myc-RXRα was identified as a PPARγ-interacting protein before [14,15]. As shown in Figure 2(B), Flag-PPARγ did not pull down Myc-HNF4α (Figure 2(B), lane 1 vs. lane 7), nor did Flag-EGFP, Flag-EGFP also failed to pull down Myc-RXRγ (lane 5 vs. lane 11) and Myc-RXRα (lane 6 vs. lane 12). However, Myc-RXRγ (lane 2 vs. lane 8) and Myc-RXRα (lane 3 vs. lane 9) was significantly pulled down by Flag-PPARγ. These experiments demonstrated the interaction between PPARγ and RXRγ.

### RXRγ increases PPARγ-mediated transcriptional activities

**Figure 3.**
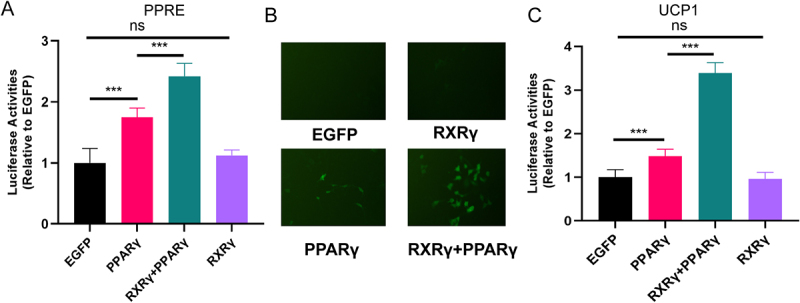
RXRγ enhances PPARγ-mediated transcriptional activities and UCP1 promotor transcription activity in a PPARγ dependent manner. (A) enhancement of PPARγ-mediated transactivation by RXRγ. HEK293 cells were transfected with a PPRE reporter and indicated expression vectors. (B) plasmids with EGFP-tagged UCP1 (EGFP-UCP1, green) were co-transfected into HEK293 cells with indicated expression vectors and photographed 48 hours post-transfection under a fluorescence microscope. (C) enhancement of UCP1 promotor transcription activity by RXRγ. HEK293 cells were transfected with a UCP1 reporter and indicated expression vectors. Data are shown as mean ± SEM (*n* = 6); ***p < 0.001.

To examine the influence of RXRγ on PPARγ-mediated transcriptional activities, a reporter assay was performed in HEK293 cells. In this assay, a PPRE reporter construct was co-transfected with various RXRγ variants and PPARγ. As illustrated in Figure 3(A), the overexpression of PPARγ resulted in a significant increase in luciferase activity (1.6-fold, *p* < 0.001 compared to the control group), and the simultaneous expression of RXRγ further augmented the luciferase activity (2.1-fold, *p* < 0.001 compared to the PPARγ group). Notably, the overexpression of RXRγ alone did not lead to an increase in luciferase activity. These findings suggest that RXRγ enhances PPRE reporter activities in a manner dependent on PPARγ.

### RXRγ enhances UCP1 promotor transcription activity in a PPARγ dependent manner

To identify the impact of RXRγ on the transcription activity of UCP1, a reporter assay was conducted in HEK293 cells wherein a UCP1 reporter construct was co-transfected with RXRγ variants and PPARγ. As shown in Figure 3(B) and C, overexpression of PPARγ significantly increased the luciferase activity (1.5-fold, *p* < 0.001 compared to the control group), and co-expression with RXRγ further enhanced the luciferase activity (3.4-fold, *p* < 0.001 compared to the PPARγ group). Importantly, overexpression of RXRγ alone did not increase the luciferase activity. This experiment indicated that RXRγ enhances UCP1 promotor transcription activity in a PPARγ dependent manner.

### RXRγ increased browning in adipocyte differentiation

**Figure 4.**
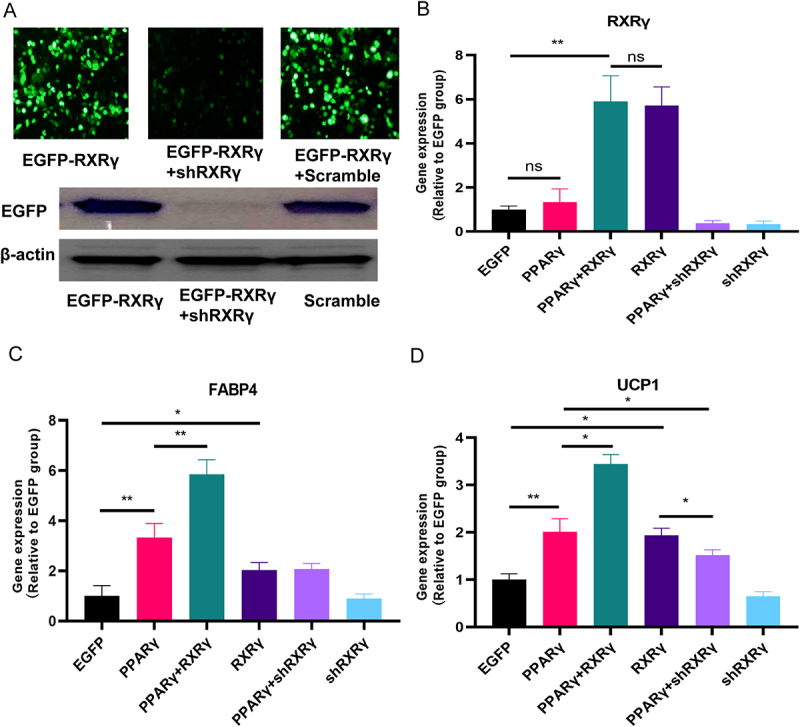
Gene expression analyses in stromal vascular cells (SVC) infected with lentiviruses as indicated. (A) validation of RXRγ suppression by shRxrγ. Plasmid EGFP-RXRγ was transfected either alone or with shRxrγ or scramble control construct in HEK293 cells for 48 hrs. The representative images of the cells under fluorescence microscopy are shown in the upper panel and western blotting for EGFP and β-actin is in the lower panel. (B)-(C) quantitative analysis illustrating the relative gene expression levels of RXRγ, FABP4, and UCP1 in SVC. Data are shown as mean ± SEM (*n* = 3). **p < 0.01; *p < 0.05.

To understand the functional impact of RXRγ on adipogenesis, depletion and overexpression studies were conducted in human preadipocytes (adipose stromal vascular cells (SCVs)). To demonstrate the specificity of the shRNA against RXRγ, HEK293 cells were infected with plasmid EGFP- RXRγ either alone or with shRXRγ or scramble control construct for 48 hours. As shown in Figure 4(A), we found that the cells infected with EGFP-RXRγ alone exhibit robust green fluorescence, indicating strong expression of the EGFP-tagged RXRγ protein. While examining EGFP-RXRγ +shRXRγ the fluorescence intensity is markedly reduced, suggesting successful knockdown of RXRγ expression by shRNA. Quantitative analysis shows that the knockdown efficiency of EGFP-RXRγ by shSTK38 was about 93%. In Western blot analysis we also found out in shRXRγ the intensity of the band is decreased compared to the EGFP control, which confirms the reduction of RXRγ protein levels upon shRNA treatment.

n the subsequent experiment, human preadipocytes, also known as adipose stromal vascular cells, were transduced with lentiviruses encoding EGFP, shRXRγ, or RXRγ, with or without PPARγ, and subsequently induced to undergo adipogenic differentiation. Quantitative PCR analyses were conducted to assess the gene expression levels of RXRγ, the adipocyte marker fatty-acid binding protein 4 (FABP4), and the browning marker UCP1. As illustrated in Figure 4B, RXRγ expression was effectively depleted by shRXRγ and overexpressed by RXRγ. Additionally, as depicted in Figure 4C, overexpression of PPARγ resulted in a 3.3-fold increase in adipogenesis, while RXRγ overexpression led to a 2.0-fold enhancement compared to baseline levels. Co-expression of RXRγ with PPARγ further augmented adipogenesis by 5.8-fold (*p* < 0.05 compared to PPARγ overexpression alone), whereas RXRγ knockdown significantly reduced PPARγ-mediated adipogenesis to 2.0-fold (*p* < 0.05 compared to PPARγ overexpression alone). These results suggest a facilitative role of RXRγ in PPARγ-mediated adipogenesis. Similarly, as shown in Figure 4D, overexpression of PPARγ or RXRγ significantly increased UCP1 expression by 2.0-fold and 1.4-fold, respectively, with a synergistic effect observed at 3.4-fold (*p* < 0.05 compared to PPARγ overexpression alone). In the comparative analysis of the RXRγ and PPARγ+shRXRγ groups, the expression levels of FABP4 were found to be comparable, suggesting similar adipogenic activity in both groups. However, the expression of UCP1 was significantly elevated in the RXRγ group (1.9-fold compared to 1.4-fold, *p* < 0.05), indicating enhanced browning in the RXRγ group. This finding demonstrates that RXRγ specifically promotes UCP1 expression. These results suggest that RXRγ plays a pivotal role in regulating adipogenesis in SVCs, and that the knockdown of RXRγ disrupts this regulatory enhancement, underscoring its significance in this pathway. Furthermore, RXRγ is identified as a key factor in promoting browning during adipocyte differentiation, which is crucial for lipid metabolism and thermogenesis.

## Discussion

Activating thermogenic adipose tissues, like BAT and beige adipose tissues could be a promising strategy to combat obesity and thus obesity-related diabetes [5,6]. Therefore, Understanding the molecular mechanisms underlying adipose tissue function is therefore critical for developing effective treatments. In this study, we observed that RXRγ is highly expressed in BAT, consistent with previous findings [16,17]. To identify further the function of RXRγ in regulating adipogenic differentiation, we performed co-immunoprecipitation analysis and found that RXRγ could be precipitated by PPARγ. Additionally, we found that the interaction between RXRγ and PPARγ promoted the transcriptional activity of PPRE and UCP1 in HEK293 cells by luciferase assay. Furthermore, we found RXRγ increased adipogenesis and browning in SCV differentiation. These results showed RXRγ is a novel PPARγ-cofactor that promotes the formation of brown adipose.

PPARγ is a member of the nuclear receptor superfamily and is activated by endogenous fatty acids as well as synthetic thiazolidinediones, such as pioglitazone and rosiglitazone, which have demonstrated clinical efficacy in treating diabetes primarily through their actions in adipose tissues [16]. Upon activation, PPARγ forms a heterodimer with RXRs, which binds to PPREs in the promoter regions of target genes [17]. PPARγ can function as a permissive partner of RXR, meaning that the PPAR/RXR heterodimer can be activated by either RXR or PPAR ligands, with simultaneous binding of both RXR and PPAR ligands leading to additive or synergistic effects [15,18], aligning with our finding that RXRγ increases PPRE transcriptional activity in the presence of PPARγ. Our study found that RXRγ was higher expressed in BAT, aligning with existing research demonstrate a selective enrichment of RXRγ in brown and beige adipose tissues. In a previous study, RXRγ has been characterized as a marker of BAT, exhibiting significantly higher expression in human perirenal (BAT-like) fat compared to subcutaneous WAT [19]. Moreover, in cultured human adipocytes subjected to a browning stimulus, RXRγ expression was observed to increase and has been described as a‘unique isoform associated with brown adipogenesis’ [20,21]. In contrast, RXRα is expressed ubiquitously across adipose depots and serves broadly as the canonical heterodimer partner of PPARγ [15,22]. In alignment with this characterization, adipocyte-specific deletion of RXRα has been shown to impair both adipogenesis and lipolysis, underscoring its essential role in adipose tissue development, while also indicating that RXRγ alone is insufficient for functional compensation [23]. This finding can explain that both RXRα and RXRγ interacted with PPARγ, as evidenced by our co-immunoprecipitation experiments (Figure 2B).

RXR is a critical member of the nuclear hormone receptor superfamily, playing a role in various physiological processes. Among the three isoforms of RXR (α, β, and γ), RXRγ is more highly expressed in BAT compared to white adipose tissue (WAT), highlighting its role in the ‘browning’ of adipocytes. RXRs possess a canonical domain structure, including DNA-binding, ligand-binding, and regulatory molecule-binding domains [18,24]. Our findings show that RXRγ enhanced transcriptional activity of PPRE and UCP1 in a PPARγ dependent manner in the HEK293 cells, indicating that RXRγ functions through its interaction with PPARγ, as previously suggested [25]. Notably, during adipocyte differentiation of SCV, overexpressing RXRγ with PPARγ promotes adipogenesis more effectively than overexpressing RXRγ or PPARγ alone, indicating that RXRγ regulates adipogenesis through its synergistic effect with PPARγ. Additionally, in the group of RXRγ and PPARγ+shRXRγ, while the expression of FABP4 were similar between the two groups, the expression of UCP1 were significantly higher in the RXRγ group, suggesting that RXRγ specifically promotes ‘browning’, which are important for lipid metabolism and thermogenesis.

Notably, RXRγ alone did not enhance UCP1 transcriptional activity in the reporter assay conducted in HEK293 cells (Figure 3C), yet it did increase UCP1 expression during SCV differentiation (Figure 4D). This discrepancy is likely due to the availability of ligands for RXRγ. As a nuclear receptor, RXRγ has been shown in several studies to significantly promote the expression of thermogenic genes, including UCP1, upon activation. For instance, the pan-RXR agonist LG100268 has been demonstrated to significantly elevate UCP1 mRNA expression in brown adipose tissue (BAT) by approximately threefold in rodent models [26]. Similarly, bexarotene (LGD1069), an RXR-selective agonist approved for clinical use, has been reported to enhance brown adipocyte differentiation and increase both BAT mass and thermogenic gene expression, including UCP1, in vivo [27]. In contrast, PPARγ agonists, such as rosiglitazone, often exhibit limited efficacy in inducing UCP1 expression. In one study, rosiglitazone did not upregulate UCP1 in BAT, whereas RXR agonists demonstrated a strong induction [28]. Additionally, two separate studies indicated that the combined activation of RXR and PPARγ (e.g. rosiglitazone plus LG100268) significantly enhances UCP1 expression beyond the effects observed with either agonist alone [29,30]. In HEK293 cells lacking agonists, RXRγ was unable to activate the UCP1 promoter. Conversely, in differentiating SCV adipocytes, the presence of endogenous or exogenous ligands, such as rosiglitazone included in the standard differentiation cocktail, likely created the requisite ligand environment for RXRγ overexpression to enhance UCP1 expression. These results indicate that RXR activation plays a distinct and potent role in promoting thermogenic gene programs.

Previous studies have demonstrated that RXRγ is crucial for fat differentiation, particularly in the late stages of adipocyte differentiation, where it forms a heterodimer with PPARγ. This association is enriched near the 5’-region of the transcriptional start site, promoting the upregulation of genes associated with fatty acid and lipid metabolism [21,29]. RXRγ has also been linked to the regulation of adipose differentiation-associated protein, which accelerates neutral lipid accumulation [31], and contributes to familial combined hyperlipidaemia [32].

Moreover, it is well established that both ageing and obesity impair thermogenic capacity, particularly by suppressing brown and beige adipocyte function [33,34]. Ageing adipose tissue is marked by a state of low-grade chronic inflammation-often termed ‘inflammaging’ that blunts UCP1 expression, impairs adipogenesis, and reduces the browning response to external stimuli such as cold exposure [35,36]. Similarly, obesity leads to hypertrophic adipocytes and inflammation, and in mouse models of aged, diet-induced obesity, there is a significant reduction in BAT UCP1 levels and thermogenic gene expression [37,38]. At the molecular level, these physiological changes are accompanied by dysregulation of PPARγ activity. For instance, PPARγ1 expression is reduced in visceral fat from individuals with severe obesity [39], and PPARγ becomes hyperacetylated in aged or obese adipocytes, a modification that diminishes its transcriptional activity and promotes BAT whitening [40]. Since RXRγ functions as a heterodimeric partner of PPARγ in adipose tissue, it is likely that these age/obesity-associated impairments in PPARγ would undermine RXRγ-PPARγ target gene activation, including UCP1. Although few studies have directly examined RXRγ in ageing or obesity, relevant evidence includes the finding that RXRγ-knockout mice are protected against high-fat diet – induced weight gain, implying that RXRγ may normally support lipid accumulation in obesogenic environments [41]. These findings collectively indicate that RXRγ-PPARγ signalling is context-dependent: while it may promote beige/brown gene programs under basal or inducible conditions, it is likely disrupted in ageing or obese states due to inflammation, altered expression, and post-translational modifications. Our investigation into RXRγ and UCP1 expression during human preadipocyte differentiation confirms that RXRγ increases UCP1 expression by forming a heterodimer with PPARγ and binding to PPRE, which is characteristic of adipocyte ‘browning’. Thus, our study identifies RXRγ as a key target for the treatment of metabolic diseases such as obesity and diabetes.

In summary, our research highlights RXRγ as a novel PPARγ cofactor that promotes ‘browning’ during SVCs differentiation by enhancing UCP1 transcription. This discovery offers new insights into PPARγ biology and its regulation, and suggests that RXRγ could be a valuable target for addressing metabolic diseases.

## Materials and methods

### Materials

Rosiglitazone was obtained from Alexis Biochemicals (San Diego, CA, USA). Protease inhibitor cocktail was sourced from Roche Applied Biosciences (Indianapolis, IN, USA). LipoD293 was purchased from Signagen Laboratories (Gaithersburg, MD, USA). Anti-Flag M2 magnetic beads, anti-Flag antibody, and 4,6-diamidino-2-phenylindole (DAPI) were acquired from Sigma-Aldrich (St. Louis, MO, USA). Anti-Myc antibody was obtained from Cell Signaling Technology (Danvers, MA, USA).

### Gene expression analysis of RNA-seq

The dataset (GSE113764) was obtained from the GEO database in MINiML format, encompassing all platforms, samples, and comprehensive GSE records within the GSE. Detailed information about the dataset can be accessed at https://www.ncbi.nlm.nih.gov/geo/query/acc.cgi?acc=GSE113764 and the original article [42]. The normalization of the data was performed using the ‘normalize’ function from the DESeq2 package in R. Based on the platform’s annotation information, probe IDs were converted to gene symbols, probes corresponding to multiple genes were excluded, and the average expression value was calculated for genes associated with multiple probes. The differences of gene expression between WAT and BAT was assessed using the Wilcoxon Matched-Pairs Signed-Ranks Test. The RNA-seq data analysis was conducted utilizing R software (version 4.2.0).

### Plasmid construction

The 3xPPRE-tk-Luc and UCP1 reporter plasmid, along with Renilla luciferase, were procured from Promega (Madison, WI, USA). The PPRE was amplified using the forward primer 5‘-atcggatccgaattcgcggccgcgttgtaaaacgacggccagtgc-3‘ and the reverse primer 5‘-tggcgtcttccatggccatggtaccaacagtaccggaatgccaagc-3‘. Similarly, the promoter of UCP1 was amplified using the forward primer 5‘-tccgaattcgcggccgctcccagtggtggctaatgaga-3‘ and the reverse primer 5‘-tcttccatggccatggtaccttgctcttcacgcctgtccgc-3ʹ. DNA sequences for triple Flag tag (referred to as ‘Flag’), triple Myc tag (referred to as ‘Myc’), and EGFP were fused to the N-terminus of EGFP, PPARγ, HNF4α, PRMT5, TCP1, MEP50, STK38, STK38L, RXRα or RXRγ by PCR using Phusion polymerase (New England Biolabs, Ipswich, MA) and subsequently cloned into a pENTR1A vector. For shRXRγ construction, primers 5‘- acctcgagggaagctgtgcaagaagatcaagagtcttcttgcacagcttccctctt-3‘ (forward) and 5‘-caaaaagagggaagctgtgcaagaagactcttgatcttcttgcacagcttccctcg-3‘ (reverse) were annealed and cloned into a pENTR1A vector containing the psiRNA-h7SK cassette (InvivoGen, San Diego, USA) using Gibson cloning. All inserts were verified for sequence fidelity by Sanger sequencing. The pENTR1A vectors with inserts were then converted into lentiviral pSMPUW vectors (Cell Biolabs, San Diego, CA, USA) via Gateway cloning.

### Cell culture, lentivirus production, and PPARγ interacting protein isolation and identification

HEK-293 (CRL-1573, ATCC, Manassas, VA) and HEK293T (CRL-3216, ATCC) cells were cultured in DMEM (Dulbecco’s Modified Eagle’s Medium) supplemented with 10% foetal bovine serum (FBS, Gibco Life Technologies), 100 µg/ml penicillin, and 100 µg/ml streptomycin (Gibco Life Technologies) for plasmid transfection and viral production. For lentivirus production, the transfer vector was co-transfected with the packaging vector pCD/NL-BH*DDD (Addgene #17,531) and the envelope vector pCMV-VSVG (Cell Biolabs) using LipoD293 in HEK-293T cells, as previously described [20,43]. To isolate PPARγ-interacting proteins, approximately 5 × 10^^^7 adipose SCVs [44,45], which had been validated for efficient differentiation into adipocytes upon lentiviral PPARγ infection, were plated in DMEM containing 10% FBS in a 10 cm dish. When cells reached 70% confluency, they were transduced with lentiviruses expressing Flag-EGFP or Flag-PPARγ. Twenty-four hours post-transduction, the cells were treated with rosiglitazone (1 µM) and insulin (70 nM) for an additional 24 hours. Following treatment, cells were washed with 1x PBS and lysed in binding buffer (50 mM Tris, pH 7.5, 150 mM NaCl, 1% NP-40, 0.25% sodium deoxycholate, 10% glycerol, 1 mM EGTA, 1 mM PMSF, protease inhibitor cocktail). The lysates were centrifuged, and the supernatants were incubated with 20 µl of anti-Flag M2 magnetic beads overnight at 4°C. The beads were collected using a magnetic stand, washed thoroughly with washing buffer (250 mM NaCl), and the bound proteins were eluted with 200 mM Flag peptide in washing buffer. Eluted proteins were analysed using LC-tandem mass spectrometry at the Proteomics Core of the University of Maryland School of Medicine. Data were reported as the number of unique peptides matching known proteins.

### Immunoprecipitation and western blot analyses

For immunoprecipitation experiments, HEK-293T cells were seeded in 15 cm dishes and transiently transfected with 10 µg of Flag- or Myc-tagged EGFP, PPARγ, HNF4α, PRMT5, TCP1, MEP50, STK38, STK38L, RXRα or RXRγ expression vectors using LipoD293 (Signagen). Forty-eight hours post-transfection, cells were lysed in RIPA buffer. The cell lysates were then incubated overnight at 4°C with either anti-Flag beads or a combination of anti-Myc antibody and protein A resin. Following incubation, the samples were washed and dissolved in 1X sample buffer for Western blot analysis. Proteomics studies were conducted in triplicate using preadipocytes from three different subjects to minimize potential bias due to donor variation.

For Western blot analysis, cell lysates or immunoprecipitates were separated using SDS – polyacrylamide gel electrophoresis and transferred to PVDF membranes. The blots were probed with anti-Flag or anti-Myc antibodies, and alkaline phosphatase (AP)-conjugated secondary antibodies. Protein bands were visualized using the BCIP/NBT Liquid Substrate System (Sigma-Aldrich) and quantified with ImageJ (Bethesda, MD).

### Adipogenesis and RT-qPCR

For the purpose of investigating adipocyte differentiation, samples of human omental and subcutaneous adipose tissues were procured from four female patients (comprising two obese and two non-obese individuals) who were undergoing semi-elective intra-abdominal surgical procedures at the University of Maryland Medical Center. The process of collagenase digestion was employed to isolate adipocytes and stromal-vascular cells (SVCs), utilizing a final concentration of 2 mg of collagenase per gram of adipose tissue in a Krebs-Ringer bicarbonate buffer supplemented with 4% albumin and 200 nM adenosine (KRB-A). Following centrifugation at approximately 200 g for 1–2 minutes, the medium beneath the buoyant adipocytes, which contained the SVCs, was extracted and subjected to a subsequent centrifugation at 800 g for 5 minutes. The resulting SVC pellet was resuspended in KRB-A and underwent three additional washes following the same protocol. SVCs were plated in duplicates, cultured in DMEM with 10% bovine serum, 100 μg of penicillin/ml, and 100 μg of streptomycin/ml, transduced with lentiviruses of EGFP, PPARγ, RXRγ, or shRXRγ, and grown to confluency. The cells were then switched to differentiation medium containing dexamethasone (250 nM), 3-isobutyl-1-methylxanthine (500 μM), and insulin (170 nM) for 2 days. On day 3, the medium was changed to culture medium supplemented with insulin (170 nM) and left for 7 days. For RT-qPCR, total RNAs were extracted using RNeasy Lipid Tissue Mini Kit (Qiagen, Valencia, CA) with oncolumn DNase digestion (Qiagen) as described before [29]. cDNAs were synthesized using AMV Reverse Transcriptase kit (Promega) from 1 µg of total RNA. Quantitative PCR was performed on a Light Cycler 480 (Roche, Indianapolis, IN) using primers 5‘-agagcgagctgagagtgagg-3‘(forward) and 5‘-cctccaaggtgaggtcagag-3‘ (reverse) for RXRγ, 5‘-tactgggccaggaatttgac-3‘ (forward), 5‘-atgcgaacttcagtccaggt-3‘ (reverse) for FABP4, and 5‘-taaaaacagaagggcggatg-3‘ (forward) and 5‘-gtcggtccttccttggtgta-3‘ for UCP1. Primers for β-actin were described previously [20]. β-actin mRNA was used for normalization of cDNA loading as an internal control. The relative expression of the target genes compared to β-actin was determined by the 2-ΔΔCT method.

## Data analysis

Data are presented as mean ± SEM. Statistical analysis was performed using one-way or two-way analysis of variance (ANOVA) followed by post-hoc tests. Specifically, Bonferroni’s test was used for between-group comparisons, and Dunnett’s test was employed for comparisons between the test group and control. The half-life of protein degradation was calculated using the exponential one-phase decay equation. All statistical analyses were conducted using GraphPad Prism 9. Differences were considered statistically significant at a level of *p* < 0.05

## Data Availability

Kun Qian had full access to all the data in the study and take responsibility for the integrity of the data and the accuracy of the data analysis. The data presented in the manuscript are available at https://www.scidb.cn/en/s/JJb6vm.
